# Record of New Termite (Blattodea, Termitidae) Species in Togo West Africa †

**DOI:** 10.3390/insects13090841

**Published:** 2022-09-15

**Authors:** Toblie Quashie Effowe, Boris Dodji Kasseney, Abdoulaye Baïla Ndiaye, Adolé Isabelle Glitho

**Affiliations:** 1 Laboratoire d’Entomologie Appliquée, Département de Zoologie, Université de Lomé, Lomé 01 BP 1515, Togo; 2 Laboratoire de Zoologie des Invertébrés Terrestres, Département de Biologie Animale, Institut fondamental d’Afrique noire, Université Cheikh Anta Diop de Dakar, BP 206, Dakar 10700, Senegal

**Keywords:** termite systematic, morphological traits, morphometric measurements

## Abstract

**Simple Summary:**

In the sub-Saharan regions of Africa, there are many termite species, of which very few have been correctly identified and described. The large majority of these species is either wrongly identified or waiting to be found and described because of the lack of identification keys and the errors within the existing keys. One way to overcome this problem is the use of reference works that contain illustrated parts of the body of termites along with accurate measurements of the features involved in termite identification. The purpose of this study is to provide pictures of the heads of soldiers (commonly used in termite identification) along with measurements of parts of the head and leg. A total of 12 termite species were examined. Seven of these species were already described, while the other five appear to have not been described before. Ten out of the twelve species are new records for the country.

**Abstract:**

In Africa, despite their economic and ecological importance, termites are still relatively unknown. Their systematic remains uncertain, the approximate number of species for many biogeographic areas is underestimated, and there is still confusion in the identification of the species for many genera. This study combined morphological traits with morphometric measurements to determine several species collected in Togo and provided head illustrations of soldiers. Termites were sampled within the frame of transects laid in several landscapes inside three different parks including: Fosse aux Lions, Galangashie, and Fazao Malfakassa. Samples were grouped by morphospecies and measurements of part of the body (length and/or width of head, mandible, pronotum, gula, and hind tibia) were conducted. Twelve termite species including *Foraminitermes* *corniferus*, *Lepidotermes* sp., *Noditermes cristifrons*, *Noditermes* sp. 1 and *Noditermes* sp. 2, *Promirotermes holmgren infera*, *Promirotermes* sp., *Unguitermes* sp., *Amitermes evuncifer*, *A. guineensis*, *A. truncatus,* and *A. spinifer* were separated and pictured. Ten new species were added to the check list of the country, including five unidentified ones. Further studies such as biomolecular analysis should be carried out in order to clarify the status of these unknown species.

## 1. Introduction

Termites species and generic richness (particularly in the central and west part of the continent) is very important [1,2,3]. Most termite species are found in tropical forest, undisturbed savanna, and protected parks [4,5,6,7]. Although several studies have been conducted (and are still ongoing) on African termites, the diversity and the taxonomy of these termites remain poorly documented. Many species are waiting to be identified, while others have been incorrectly identified [8,9,10,11]. These taxonomical problems were pointed out by a group of researchers working on African termites [12].

In fact, the identification of African termites is based not only on the comparison of samples with reference species (which most of the time have not been correctly identified) but also with reference works by famous taxonomists [13,14,15,16,17,18,19]. These reference works combine morphological traits (shape, color of different parts of the body) and morphometric measurements (length, width, and depth) of certain parts of the body of the soldier caste. The worker caste of termites is also used for identification, and the same features as soldiers are examined or measured [20]. In addition to the external morphological traits, gut anatomy is often used to identify termites [21]. All these descriptions are sometimes illustrated by hand drawing of the whole or part of the body of the termites instead of color images. This weakness is understandable as most of these reference works were carried out and published a century ago with tools and means that did not allow color images. However, since then, very few revisions have been conducted on African termite species. Among the useful reference works, several are also written in languages such as German [15], Italian [13,14] and French [8,9,17,18], which unlike English are not easily accessible to many researchers. Fortunately, recent research on termite taxonomy, in addition to using English, also uses images of the whole or part of the body of termites [22,23]. The research on termites in Togo (a West African country) has been hampered by the abovementioned issues. The check list of termite species of the country needs to be established. Although some recent studies have been carried out on the systematization of termite species, many areas of the country have still not been prospected, and their respective species remain unknown. The purpose of this study is to contribute to the knowledge of termites species in the country and to share color images along with the morphological features and morphometric measurements of several emblematic species from the central and northern parts of Togo.

## 2. Materials and Methods

### 2.1. Study Areas

**Figure 1:**
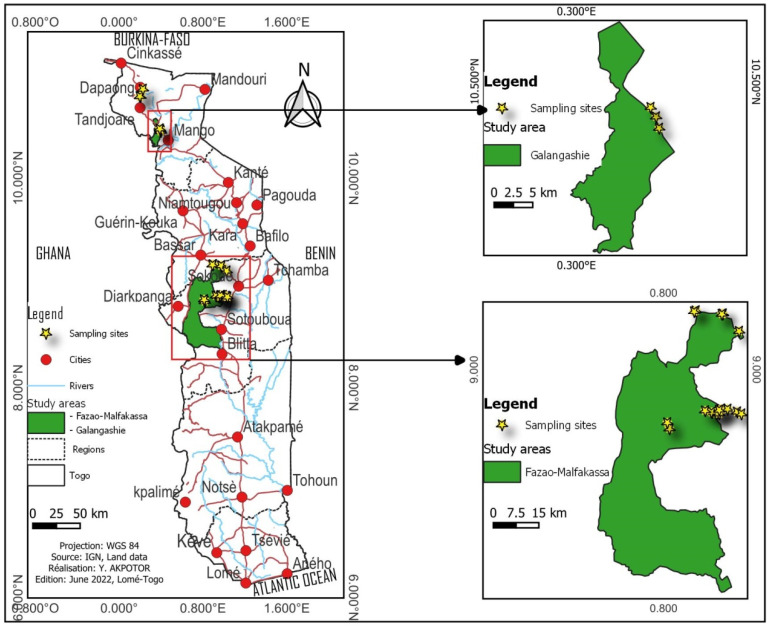
Map of Togo with prospected parks.

Termites were collected from three different parks (Figure 1) including: Fosse aux lions (10°46′–10°49′ N and 0° 11′–0°14′ E), Galangashie (10°19′–20°28′ N and 0°14′–0°27′ E), and Fazao-Malfakassa (8°20′–9°35′ N and 0°35′–1°02′ E).

Fosse aux lions and Galangashi, both located in the northern part of Togo, are characterized by a Sudanian tropical climate with a long dry season (November to May) and a long rainy season (June to October). In these two parks, the mean temperatures range from 29 ± 2 °C during the rainy season to 30 ± 3 °C during the dry season. The annual rainfall is 986 mm, and the landscape is shrubby savanna. Fazao-Malfakassa, located in the center of the country, is characterized by a semi-humid tropical climate with a rainy season from April to October and a dry season from November to March. The mean temperatures range from 27.5 ± 1.5 °C during the dry season to 27 ± 2 °C during the rainy season. The annual rainfall is 120 mm, and the landscape is composed of dry forests, gallery forests, shrubby savanna, and fallows.

### 2.2. Termites Sampling

A total of 27 sampling sites were prospected with three transects per sampling site (81 transects for the whole study). The standard protocol [24] adapted to the savanna ecosystem [4,25] was used. Each transect of 100 × 5 m was divided into 20 sampling units of 5 × 2 m, which were sampled for 15 min [26,27]. Termites were searched within the frame of each sampling unit inside mounds, litter, wood, and grasses on trees by two well-trained collectors. After this searching on the surface, termites were also searched throughout eight soil scraps.

### 2.3. Termites Identification

Morphological traits (shape of the mandibles and the position of the mandible tooth) of the soldier and the number of antennal segments were used to separate species. Measurements of head width and length, left mandible length, pronotum width, and hind tibia length were made with a stereomicroscope (Leica EZ4) equipped with an integrated camera connected to a computer. Voucher specimens are conserved in the “Laboratoire d’Entomologie” of the University of Lomé (Lomé, Togo).

### 2.4. Statistical Analysis

A total of 5 individual soldiers (when possible) were used for morphometric measurements. Thus, the morphometric data are presented as the mean of the measurements of each of chosen morphological feature from 5 individual soldiers.

Factorial discriminant analysis (using morphometric data) was used to separate species with close measurements within the same genus. XLSTAT (version 6.1.9. 2003 Addinsoft, Inc., Broklyn, NY, USA) software was used for the factorial discriminant analysis.

## 3. Results

Twelve termite species belonging to seven genera and three subfamilies (Table 1) were examined in this study. All these species belonged to the Termitidae family. Except for *A. evuncifer* and *A. guineensis*, the other 10 species were recorded for the first time in Togo.

### 3.1. Foraminitermes Species

**Figure 2:**
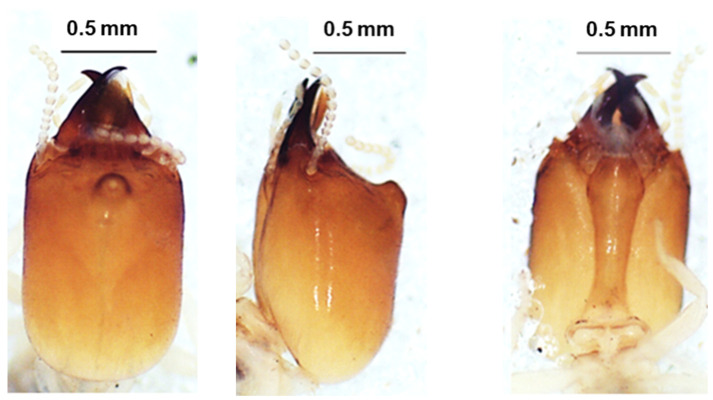
The head of *Foraminitermes corniferus* soldier in dorsal view (**left**), lateral view (**middle**), and ventral view (**right**).

The head of the soldier was yellow-brown and sub-rectangular in the dorsal view (Figure 2). The labrum with a whitish tip was a bit shorter than the mandibles, which were shorter than the head capsule. The mandibles were brown at the base but darker at the top. The antennae had 15 articles. The morphometric measurements of this species are given in Table 2.

### 3.2. Lepidotermes Species

**Figure 3:**
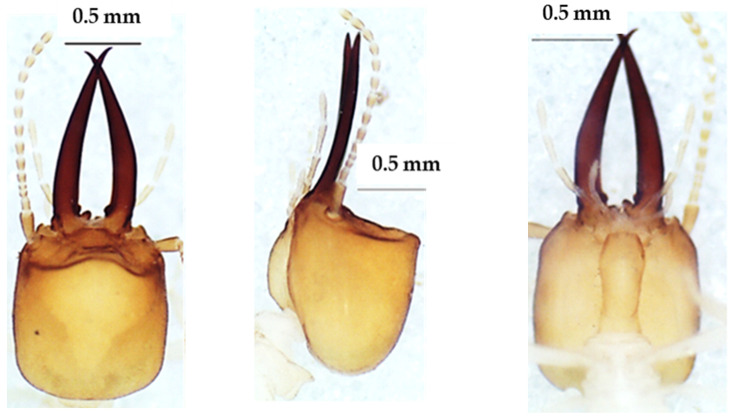
The head of *Lepidotermes* sp. soldier in dorsal view (**left**), lateral view (**middle**), and ventral view (**right**).

The head in the dorsal view (Figure 3) was almost square. The mandibles were wider at the base but tapered at the top. Each mandible had a basal tooth. There were 14 antennal articles. The morphometric measurements of this species are presented in Table 3.

### 3.3. Noditermes Species

**Figure 4:**
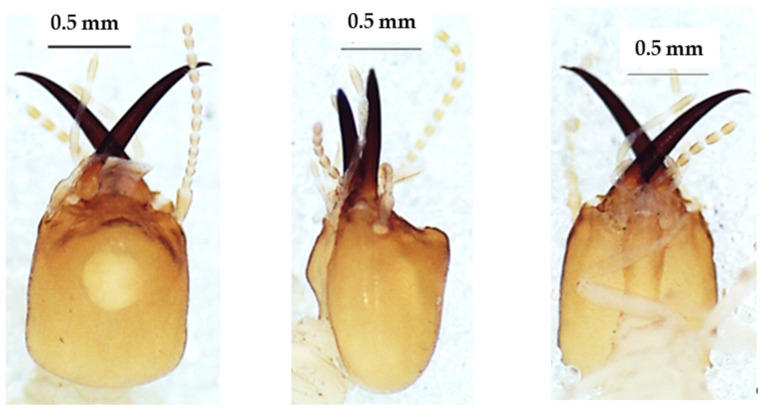
The head of *Noditermes cristifrons* soldier in dorsal view (**left**), lateral view (**middle**), and ventral view (**right**).

**Figure 5:**
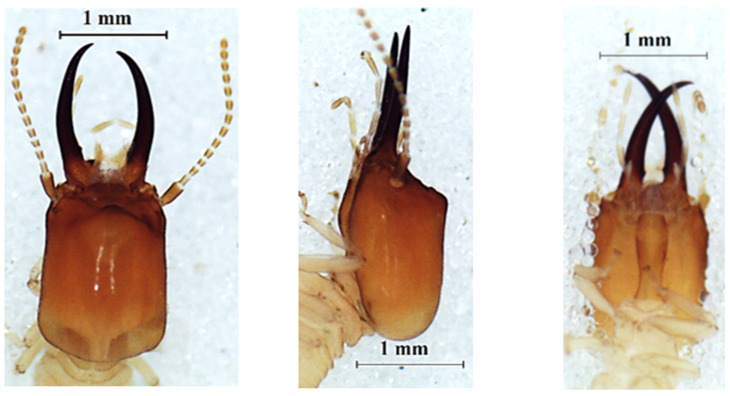
The head of *Noditermes* sp. 1 soldier in dorsal view (**left**), lateral view (**middle**), and ventral view (**right**).

**Figure 6:**
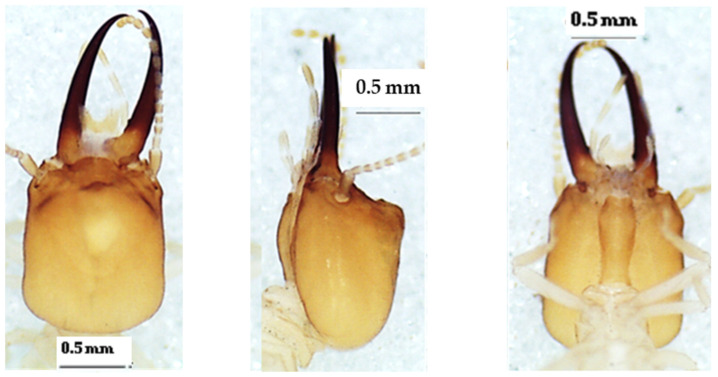
The head of *Noditermes* sp. 2 soldier in dorsal view (**left**), lateral view (**middle**), and ventral view (**right**).

**Figure 7:**
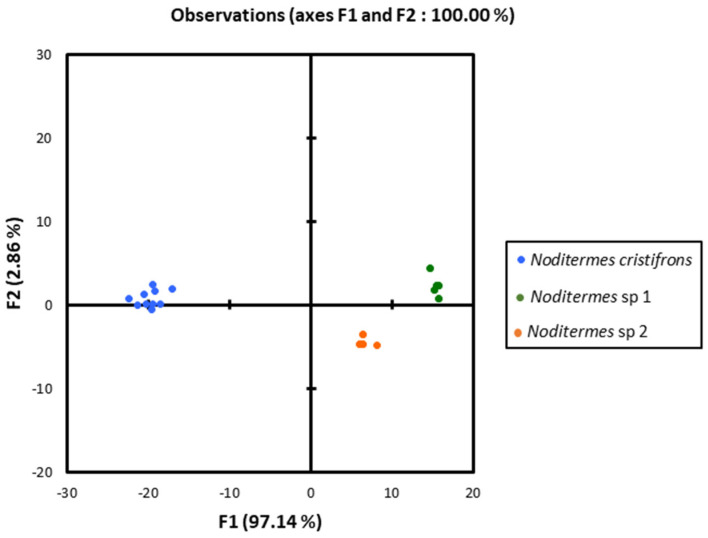
*Noditermes* species discrimination by factorial discriminant analysis.

The heads of these three species in dorsal view (Figure 4, Figure 5 and Figure 6) were almost rectangular and orange. Their respective labrum was bifurcate at the top. There were 14 antennal segments. However, they were distinct species, and their differences are highlighted in Table 4, Table 5 and Table 6. The head capsule of *Noditermes* sp. 1 was larger (1.626 ± 0.027 mm) and wider (1.15 ± 0.046 mm) than the other two *Noditermes*. Similarly, the mandibles of *Noditermes* sp. 1 were also longer (1.35 ± 0.026 mm) than those of the two other species. *N. cristifrons* had the smaller pronotum (0.508 ± 0.008 mm). The factorial differential analysis (Figure 7) shows clearly that these three *Noditermes* species were distinct.

### 3.4. Unguitermes Species

**Figure 8:**
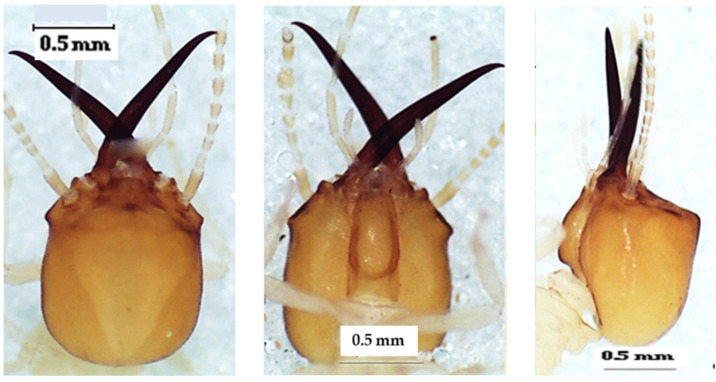
The head of *Unguitermes* sp. soldier in dorsal view (**left**), lateral view (**middle**), and ventral view (**right**).

The head of the soldier in the dorsal view (Figure 8) was almost square and yellow-orange. The top of the labrum was rectilinear and wider than the base. The mandibles were longer than the head capsule (Table 7). There were 14 antennal articles.

### 3.5. Amitermes Species

**Figure 9:**
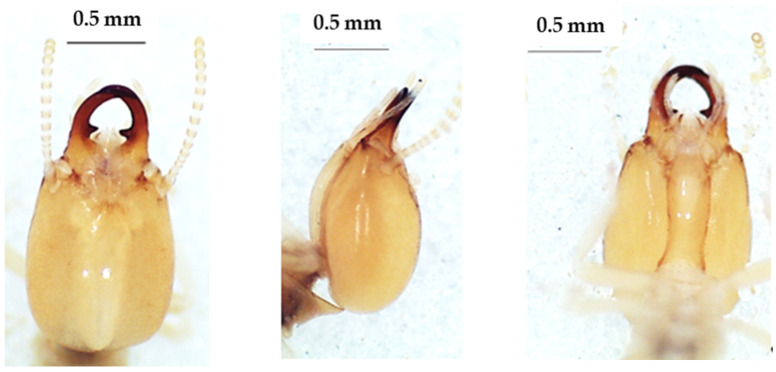
The head of *Amitermes evuncifer* soldier in dorsal view (**left**), lateral view (**middle**), and ventral view (**right**).

**Figure 10:**
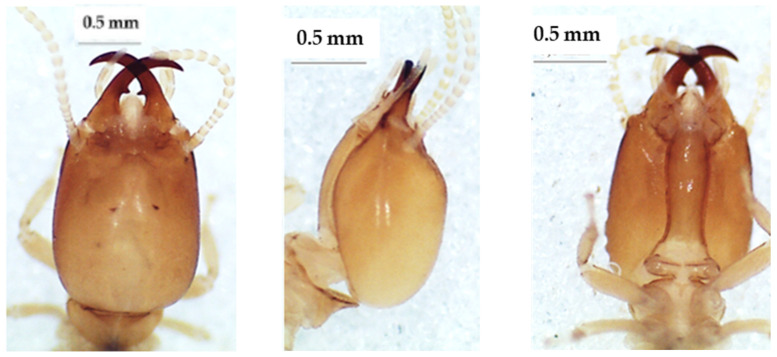
The head of *Amitermes guineensis* soldier in dorsal view (**left**), lateral view (**middle**), and ventral view (**right**).

**Figure 11:**
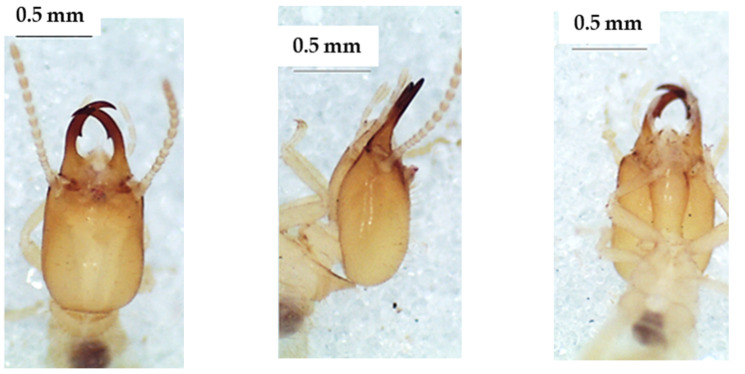
The head of *Amitermes spinifer* soldier in dorsal view (**left**), lateral view (**middle**), and ventral view (**right**).

**Figure 12:**
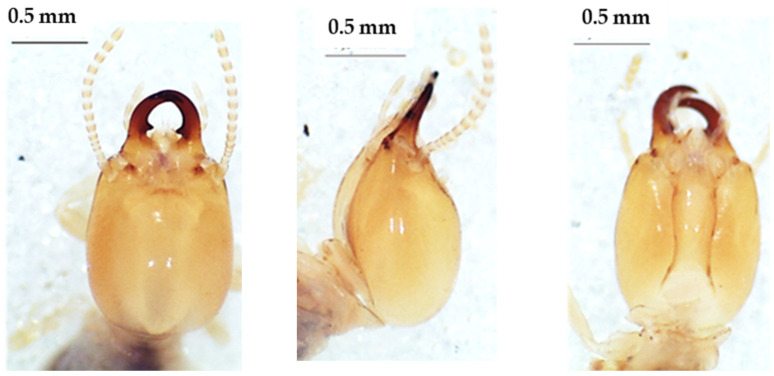
The head of *Amitermes truncatidens* soldier in dorsal view (**left**), lateral view (**middle**), and ventral view (**right**).

**Figure 13:**
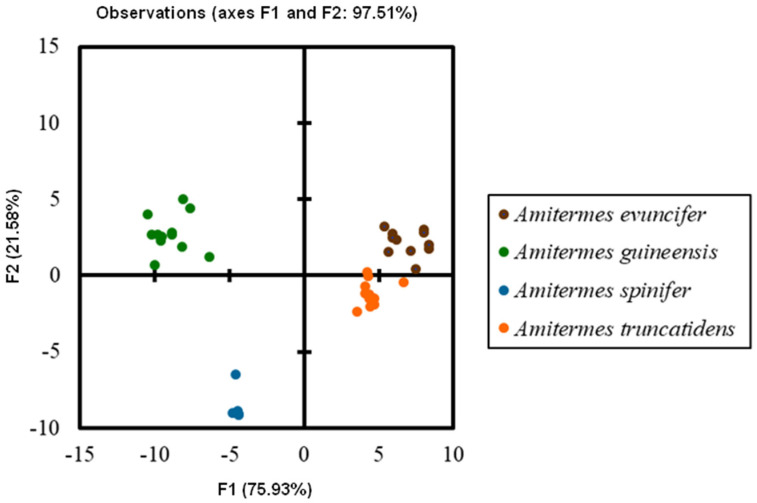
*Amitermes* species discrimination by factorial discriminant analysis.

The four species of *Amitermes* including *A. evuncifer* (Table 8, Figure 9), *A. guineensis* (Table 9, Figure 10), *A. spinifer* (Table 10, Figure 11), and *A. truncatidens* (Table 11, Figure 12) were unambiguously identified. Apart from *A. spinifer* (with 13 antennal segments), the soldiers of the other three species had 14 antennal segments. All four species had mandibles strongly curved at the top, and each mandible had a tooth in its inner side. The tips of the mandibular teeth of *A. evuncifer*, *A. truncatidens*, and *A. guineensis* were horizontal, whereas in *A. spinifer* the tips of the mandibular teeth were pointing down. *A. guineensis* differed from the other species by the rectangular shape of the head capsule and especially by the average length, which was greater (1.225 ± 0.031 mm) than those of the other species. The species with a shorter head capsule was *A. spinifer* (0.933 ± 0.018 mm). The left mandible of *A. guineensis* was the longest (0.722 ± 0.058 mm), while *A. truncatidens* had the shortest left mandible (0.547 ± 0.023 mm). The ranges and measurements of head length, head width, left mandible length, pronotum width, gula width, and hind tibia length for each species are presented in Table 8, Table 9, Table 10 and Table 11, respectively. Although the *A. evuncifer* and *A. truncatidens* measurements were close (Table 8 and Table 11), the factorial discrimant analysis showed that they were separate species (Figure 13), as well as the other two species (*A. guineensis* and *A. spinifer*).

### 3.6. Promirotermes Species

**Figure 14:**
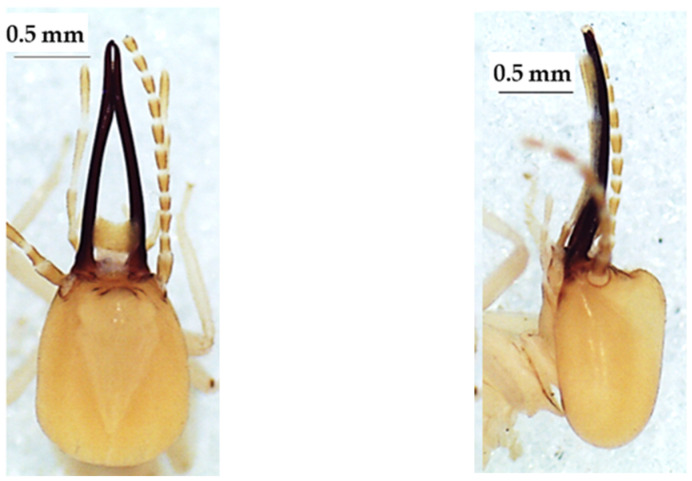
The head of *Promirotermes holmgreni infera* soldier in dorsal view (**left**) and ventral view (**right**).

**Figure 15:**
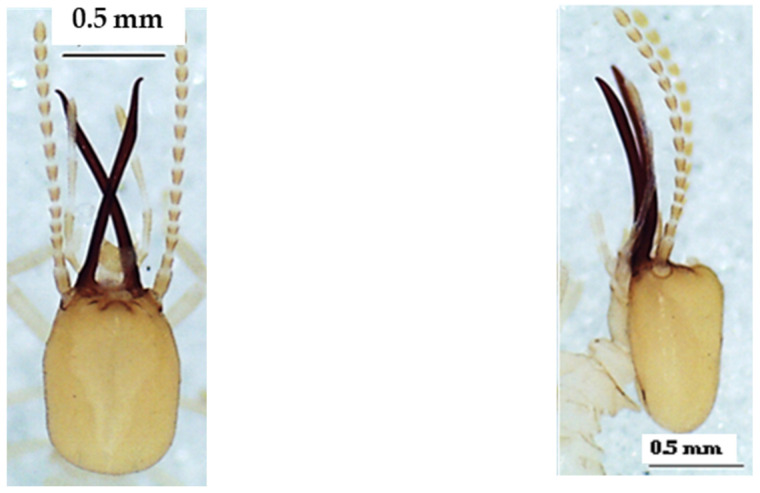
The head of *Promirotermes* sp. soldier in dorsal view (**left**) and ventral view (**right**).

For the two species of this genus, *P. holmgreni infera*, (Table 12, Figure 14) and *Promirotermes* sp. (Table 13, Figure 15) the hind part of the head was wider than the front part. The maxillary palps of the two species were as long as the mandibles, which were tapered at the top. Their respective labra were bifurcate and wider at the top.

## 4. Discussion

Among the recorded species, several had already been described, while others seemed to be ambiguous, because their measurements did not fall within the range of known species from West Africa. Foraminitermitinae include three genera: *Foraminitermes*, *Labritermes*, Holmgren 1914 and *Pseudomicrotermes*, Holmgren 1912 [28,29]. *Foraminitermes* species have been revised by Krishna [30]. Among the six described species of *Foraminitermes*, two species including *F. tubifrons* Holmgren 1912 and *F. valens* Silvestri 1914 [13], were both recorded in West Africa (Guinea, Ivory Coast, and Nigeria) and neighboring countries (Cameroon and Congo). *F. corniferus* is close to *F. valens* for some morphometric values in comparison to other species. However its head is larger than that of *F. valens*. As the other five *Foraminitermes* species, *F. corniferus* is endemic to the Ethiopian zoogeographical region and was recorded in Congo (Mukimbungu) [30]. The occurrence of *F. corniferus* in our study area indicates that the distribution area of this species, hitherto known from the Congo (Mukimnungu), extends to Togo. The genus *Lepidotermes* contains nine described species [29]. All these species are found principally in southern Africa [30,31,32,33,34]. Among these species, *Lepidotermes* sp. is morphologically close but smaller than the *Lepidotermes lounsburyi* and *Lepidotermes planifacies* described respectively by Silvestri [14] and Williams [35]. *N. cristifrons,* previously described as *Cubitermes cristifrons* [36], seemed to be the sole species of the seven described of the *Noditermes* genus [29] to occur in West Africa. It was recorded in Gambia in a forest ecosystem. The other two undetermined *Noditermes* (*Noditermes* sp. 1 and sp. 2) were all larger than *N. cristifrons* and appeared to not yet be described. This appears to be the same for *Unguitermes* sp. which was smaller than *Unguitermes acutifrons* [14] and *Unguitermes magnus*, Ruelle 1973 [37]. All the representative castes (imago, soldiers, and workers) of the four *Amitermes* species were already described and are all found in the Ethiopian zoogeographical region [19]. In this study, the ranges and means of the measurements of the soldiers fell within the ranges and means of respective species. *Amitermes spinifer* had the shorter and smaller head of all, while *A. guineensis* had the longer and the larger one. Compared to *Promirotermes holmgren holmgren*, *P. holmgren infera*, and *P. holmgren redundans,* the known species from West Africa, the *Promirotermes* sp. presented was clearly smaller and different by the shape of its head.

## 5. Conclusions

Twelve termite species were partially (head) illustrated in our study. Seven of these species including the four species of *Amitermes* genus (*A. evuncifer*, *A. guineensis*, *A. spinifer,* and *A. truncatus*), *Foraminitermes corniferus*, *Noditermes cristifrons,* and *Promirotermes holmgren inferea* were already described. The other five (*Lepidotermes* sp., *Noditermes* sp. 1, *Noditermes* sp. 2, *Unguitermes* sp., and *Promirotermes* sp.) were different by their measurements from the known species of the respective genus. This study was the first in Togo to present termite species with measurements and illustrations. It can be used as reference work for future taxonomic research.

## Figures and Tables

**Table 1:** Termite species examined.

Subfamily	Species	Distribution
Foraminitermitinae Holmgren, 1912	*Foraminitermes corniferus* (Sjöstedt, 1905)	Fazao-Malfakassa
	*Lepidotermes* sp.	Fazao-Malfakassa
	*Noditermes cristifrons* (Wasmann, 1911)	Fazao-Malfakassa, Galangashi
Cubitermitinae Weidner, 1956	*Noditermes* sp. 1	Fazao-Malfakassa, Galangashi
	*Noditermes* sp. 2	Fazao-Malfakassa, Galangashi
	*Unguitermes* sp.	Fazao-Malfakassa
	*Amitermes evuncifer* (Silvestri,1912)	Fazao-Malfakassa, Galangashi, Fosse aux lions
	*Amitermes guinensis* (Sands, 1992)	Fazao-Malfakassa, Galangashi, Fosse aux lions
Termitinae Latreille, 1802	*Amitermes spinifer* (Silvestri, 1914)	Galangashi, Fosse aux lions
	*Amitermes truncatidens* (Sands, 1959)	Fazao-Malfakassa, Galangashi, Fosse aux lions
	*Promirotermes holmgreni infera* Silvestri, 1914	Fazao-Malfakassa
	*Promirotermes* sp.	Fazao-Malfakassa

**Table 2:** The measurements (mm) of the soldiers of *Foraminitermes corniferus*.

Measured Characters	Measurements (mm)
Head length	1.28
Head width	0.853
Left mandible length	0.555
Pronotum width	0.562
Gula width	0.817
Hind tibia length	0.7

**Table 3:** The measurements (mm) of the soldiers of *Lepidotermes* sp.

Measured Characters	Measurements (mm)
Head length	1.14
Head width	1.05
Left mandible length	1.29
Pronotum width	0.482
Gula width	0.638
Hind tibia length	0.741

**Table 4:** The measurements (mm) of the soldiers of *Noditermes cristifrons*.

Measured Characters	Range (mm)	Mean ± SD
Head length	1.22–1.34	1.268 ± 0.038
Head width	0.91–1.09	1.002 ± 0.059
Left mandible length	1.17–1.38	1.237 ± 0.082
Pronotum width	0.5–0.516	0.508 ± 0.008
Gula width	0.239–0.31	0.279 ± 0.025
Hind tibia length	0.754–0.878	0.791 ± 0.044

**Table 5:** The measurements (mm) of the soldiers of *Noditermes* sp. 1.

Measured Characters	Range (mm)	Mean ± SD
Head length	1.6–1.67	1.626 ± 0.027
Head width	1.07–1.19	1.15 ± 0.046
Left mandible length	1.31–1.38	1.35 ± 0.026
Pronotum width	0.609–0.62	0.616 ± 0.004
Gula width	0.263–0.301	0.284 ± 0.015
Hind tibia length	0.734–0.936	0.886 ± 0.086

**Table 6:** The measurements (mm) of the soldiers of *Noditermes* sp. 2.

Measured Characters	Range (mm)	Mean ± SD
Head length	1.36–1.39	1.372 ± 0.012
Head width	1.09–1.12	1.105 ± 0.013
Left mandible length	1.26–1.33	1.285 ± 0.031
Pronotum width	0.563–0.597	0.579 ± 0.013
Gula width	0.265–0.288	0.275 ± 0.006
Hind tibia length	0.808–0.832	0.814 ± 0.012

**Table 7:** The measurements (mm) of the soldiers of *Unguitermes* sp.

Measured Characters	Range (mm)	Mean ± SD
Head length	1.16–1.2	1.18 ± 0.028
Head width	0.986–1.02	1.003 ± 0.024
Left mandible length	1.21–1.28	1.245 ± 0.045
Pronotum width	0.54–0.6	0.57 ± 0.04
Gula width	0.301–0.32	0.31 ± 0.013
Hind tibia length	0.772–0.793	0.782 ± 0.014

**Table 8:** The measurements (mm) of the soldiers of *Amitermes evuncifer*.

Measured Characters	Range (mm)	Mean ± SD
Head length	1.06–1.19	1.134 ± 0.049
Head width	0.93–0.975	0.954 ± 0.014
Left mandible length	0.594–0.709	0.660 ± 0.038
Pronotum width	0.587–0.615	0.600 ± 0.011
Gula width	0.252–0.3	0.279 ± 0.016
Hind tibia length	0.828–1.2	0.970 ± 0.126

**Table 9:** The measurements (mm) of the soldiers of *Amitermes guineensis*.

Measured Characters	Range (mm)	Mean ± SD
Head length	1.17–1.27	1.225 ± 0.031
Head width	0.677–0.979	0.871 ± 0.008
Left mandible length	0.611–0.795	0.722 ± 0.058
Pronotum width	0.139–0.287	0.438 ± 0.013
Gula width	0.139–0.287	0.213 ± 0.041
Hind tibia length	0.503–0.519	0.509 ± 0.005

**Table 10:** The measurements (mm) of the soldiers of *Amitermes spinifer*.

Measured Characters	Range (mm)	Mean ± SD
Head length	0.865–0.954	0.933 ± 0.018
Head width	0.681–0.742	0.726 ± 0.027
Left mandible length	0.585–0.624	0.613 ± 0.017
Pronotum width	0.472–0.492	0.486 ± 0.009
Gula width	0.257–0.26	0.288 ± 0.020
Hind tibia length	0.642–0.65	0.643 ± 0.017

**Table 11:** The measurements (mm) of the soldiers of *Amitermes truncatidens*.

Measured Characters	Range (mm)	Mean ± SD
Head length	1.01–1.13	1.064 ± 0.037
Head width	0.943–0.992	0.966 ± 0.016
Left mandible length	0.522–0.577	0.547 ± 0.023
Pronotum width	0.564–0.576	0.575 ± 0.007
Gula width	0.27–0.314	0.284 ± 0.021
Hind tibia length	0.756–0.816	0.785 ± 0.025

**Table 12:** The measurements (mm) of the soldiers of *Promirotermes holmgreni infera*.

Measured Characters	Range (mm)	Mean ± SD
Head length	1.27–1.31	1.29 ± 0.014
Head width	0.994–1.08	1.040 ± 0.034
Left mandible length	1.57–1.74	1.674 ± 0.063
Pronotum width	0.787–0.826	0.802 ± 0.02
Gula width	0.27–0.36	0.31 ± 0.037
Hind tibia length	1.1–1.23	1.136 ± 0.05

**Table 13:** The measurements (mm) of the soldiers of *Promirotermes* sp.

Measured Characters	Range (mm)	Mean ± SD
Head length	0.877–0.879	0.878 ± 0.014
Head width	0.692–0.995	0.693 ± 0.021
Left mandible length	1.11–1.13	1.12 ± 0.015
Pronotum width	0.75–0.77	0.76 ± 0.014
Gula width	0.33–0.344	0.337 ± 0.009
Hind tibia length	0.667–0.67	0.668 ± 0.0016

## Data Availability

The data presented in this study are available on request from the corresponding author.
